# Reduction in systemic glucocorticoid utilization among COPD patients with type 2 inflammation treated with biologics

**DOI:** 10.1186/s12890-025-03809-4

**Published:** 2025-07-10

**Authors:** Truong-An A. Ho, Stephen Dachert, Anugya Mittal, Gerard J. Criner

**Affiliations:** 1 Department of Medicine, Temple University Hospital, PA Philadelphia, USA; 2 Department of Thoracic Medicine and Surgery, Lewis Katz School of Medicine, PA Philadelphia, 19140 USA

**Keywords:** Glucocorticoid sparing, Biologic therapy, COPD, Type 2 inflammation, Eosinophilia

## Abstract

**Background:**

Systemic glucocorticoids are associated with significant side effects, however, are essential in the treatment of acute exacerbations of chronic obstructive pulmonary disease (COPD). Biologic therapies in COPD with type 2 (T2) inflammation have shown benefit in reducing exacerbations, but their impact on glucocorticoid utilization remains unclear. We aim to examine if use of biologics in COPD patients reduces glucocorticoid burden.

**Methods:**

A retrospective review of the electronic medical record (2016–2023) was performed. Patients with COPD that were treated with biologics were included. Data collected included demographics, baseline comorbidities, eosinophil count, pulmonary function testing and dispense reports for glucocorticoids. The primary outcomes were a change in the number of glucocorticoid dispenses and total cumulative systemic glucocorticoid dosage, in the year prior and post initiation of therapy.

**Results:**

56 patients (mean age 71 ± 8.5) were included in the study. 55% had coronary artery disease, 25% had heart failure, 71% had hypertension, 14% had stroke and 30% had diabetes. Biologics significantly reduced annual glucocorticoid dispenses (3.38 ± 2.58 vs. 2.22 ± 2.33, mean reduction 1.16, 95% CI 0.45–1.87, *p* = 0.002) and cumulative dosage (1073 ± 831 mg vs. 659 ± 723 mg, mean reduction 413.2 mg, 95% CI 180.8–645.6, *p* = 0.001). There was no strong association between baseline eosinophil count and glucocorticoid utilization.

**Conclusions:**

In this real-world cohort of COPD patients with T2 inflammation, the addition of biologic therapies was associated with a significant reduction in systemic glucocorticoid usage, both in terms of dispense frequency and overall total dosage of systemic glucocorticoids. This highlights the potential of biologics to reduce glucocorticoid-related adverse effects in COPD patients.

## Background

Systemic glucocorticoids are an important therapy in the acute management of chronic obstructive pulmonary disease (COPD) exacerbations. Systemic glucocorticoids have also been reported to be used on a chronic basis in about 5% of the patient population with COPD, especially those who are more symptomatic, have frequent and severe exacerbations, or have more emphysematous destruction [1]. There are significant side effects with the use of systemic steroids- either on an acute or chronic basis. Systemic glucocorticoids have adverse effects on all organ systems, with common concerns being hyperglycemia, weight gain, fluid retention, hypertension, osteoporosis and neuropsychiatric symptoms [2]. In the past decades, long term steroid utilization has decreased due to these side effects, along with studies that show a correlation between long term steroid use and mortality [3, 4]. While many associate these side effects with long term usage, even short bursts of glucocorticoids have been associated with increased negative outcomes, including risk of sepsis, venous thromboembolism and fracture [5]. Patients who have longer courses of glucocorticoids (10 vs. 5 days) have increased risk of pneumonia related hospitalization and all-cause mortality [2]. Limiting the use of systemic glucocorticoids remains challenging given that in COPD exacerbations, systemic glucocorticoids may reduce dyspnea and rates of relapse, decrease hospital length of stay and transiently improve FEV1[6–8]. This data is reflected in the current guidelines for COPD, and short term glucocorticoids (e.g., 5–10 days) are recommended in the management of acute exacerbations, and do not have a role in chronic treatment [9, 10].

Recent randomized clinical trials have shown mixed results in terms of biological therapy on COPD exacerbations but have not addressed their impact on utilization of systemic glucocorticoids. In METREX/METREO, GALATHEA/TERRANOVA, and BOREAS/NOTUS, the patients enrolled required at least two moderate COPD exacerbations, however, the studies did not quantify the amount of glucocorticoids pre and post therapy [11–14]. This is contrasted to asthma, in which there is robust data that shows that the same biologic agents can reduce glucocorticoid utilization [15–18]. In terms of COPD, data is limited to a small case series in which 7 patients on chronic daily maintenance therapy were treated with anti IL5 therapy and had reductions in oral corticosteroid usage [19]. With dupilumab’s recent FDA approval as the first biologic therapy for COPD patients, and ongoing clinical trials reassessing IL-5 agents, more studies are needed to assess the benefits and risks of biologics. We aim to further assess the ability of biologic therapies to reduce utilization of systemic glucocorticoids in a real-world population of COPD patients with T2 inflammation.

## Methods

### Study description

This is a single-center retrospective review of the electronic medical record between 1/1/2016 to 10/1/2023 for consecutive patients that were ever prescribed a biologic agent and carried a diagnosis code for COPD. This study was performed in accordance with the ethical standards of the Helsinki Declaration of 1975 and approved by the Temple University Institutional Review Board (Protocol 31820).

### Patient population

All patients > 40 years of age, who were prescribed biologic therapy, including mepolizumab, benralizumab and dupilumab were included. Patients were excluded if they had been on multiple biologics, had a history of interstitial lung disease, only had a history of asthma, or were on biologics for alternate therapies such as for dermatologic disease. Patients were also excluded if they were missing office appointments or pulmonary function tests (PFTs), one year prior and one year post initiation of therapy or if patient never started treatment.

### Data collected

Data collected included the type and number of comorbidities, prescribed inspired oxygen at rest, degree of emphysema quantitated on HRCT imaging, and peripheral blood eosinophil count at initiation and nadir.

Chart review was performed to quantify glucocorticoid utilization, specifically on dispenses for exacerbations. This was defined by association with exacerbation and excluded chronic steroid administration. This was due to the ambiguity with chronic glucocorticoid dosing documentation. The cumulative dosage of glucocorticoids, in milligrams (mg) of prednisone, was collected 1 year prior and 1 year post initiation of biologic therapy. The total number of glucocorticoids dispenses were quantified 1 year prior and 1 year post initiation of biologic therapy.

### Endpoints

The primary end point was the cumulative yearly systemic glucocorticoid utilization pre-initiation of biologics compared to post initiation. This was quantified as both the total cumulative mg of prednisone in the year, and the total number of glucocorticoids dispenses. The baseline eosinophil count was also examined to determine if there was an association with degree of glucocorticoid utilization after biologic therapy.

### Statistical analysis

Descriptive statistics were used for baseline demographics and clinical characteristics of the study population. Continuous variables are presented as mean ± standard deviation or median ± interquartile range, unless otherwise stated. Paired t-test was performed to compare systemic glucocorticoid utilization before and after biologic initiation. Linear regression was performed to assess the association between baseline blood eosinophil count and metrics of glucocorticoid utilization. Analyses were performed using SPSS version 25, New York, USA. Statistical significance was defined as a p valve less than 0.05 and a confidence interval that did not cross 1.

## Results

### Patient demographics

1,241 patients were obtained from the initial query of the electronic medical record. 668 patients remained after duplicate entries were removed. After removing patients who received biologic therapy for a diagnosis other than COPD or asthma-COPD overlap syndrome, 192 patients remained, and 56 patients who had complete pre and post initiation office visits and PFTs were included in the final analysis.

The mean (± SD) age of patients was 71 ± 8.5yrs (range 47–91 years) Patients were equally split between male and female patients (50%, *n* = 28 in each group). 37 (66.1%) were Caucasian, 10 (17.9%) were African American, 6 (10.7%) were Hispanic, and 3 (5.3%) were of another race. In terms of comorbid disease, 55.4% of patients had coronary artery disease, 25% had heart failure, 71.4% had hypertension, 14.3% had prior stroke, and 30.4% had diabetes. (Table 1). There were no active smokers, and patients had a mean of 47 pack years. Patients were on optimal medical therapy as determined by the treating physician, with 52 (92%) were on triple inhaled therapy. 25 (44.6%) of patients were receiving glucocorticoids on a chronic basis.

**Table 1:** Demographics and clinical characteristics of the patients at baseline

Variable	*N* = 56
Age, years (mean ± SD)	71 ± 8.5
Female no. (%)	28 (50)
Male no. (%)	28 (50)
Race or ethnic group no. (%)	
Caucasian/white	37 (66.1)
African american/black	10 (17.9)
Hispanic	6 (10.7)
Other	3 (5.3)
Pulmonary function tests mean ± SD	
FEV1% pred	41 ± 20
FVC % pred	75 ± 17.6
FEV1/FVC	41.6 ± 15.3
TLC % pred	107 ± 24.8
RV % pred	159 ± 48.8
DLCO % pred	42 ± 18.8
Mean pack years	47 ± 34.8
Moderate exacerbations in year prior (Mean ± SD)	3.63 ± 2.6
Severe exacerbations in year prior (Mean ± SD)	1.34 ± 1.9
Combined moderate and severe exacerbations in year prior (Mean ± SD)	4.8 ± 4.13
Treatment with triple inhaled therapy no.(%)	52 (93)
Treatment with glucocorticoids on chronic basis no. (%)	25 (44.6)
Glucocorticoid dispenses in year prior (Mean ± SD)	3.38 ± 2.58
Glucocorticoid dosage in year prior– mg of prednisone (Mean ± SD)	1072.56 ± 831.07
Eosinophils at initiation - cells/µL (Mean ± SD)	414 ± 296
CT evidence of emphysema no. (%)	40 (71.4)
Comorbid diseases no. (%)	
Coronary artery disease	31 (55.41)
Heart failure	14 (25)
Hypertension	40 (71.4)
Stroke history	8 (14.3)
Diabetes mellitus	17 (30.4)

Baseline Pulmonary Function Testing, Imaging, Exacerbations and Eosinophil Counts.

Baseline FEV1% predicted was 41 ± 20. Patients in our study on average had significant gas trapping (RV% Predicted 159 ± 48.8) and moderately reduced DLCO % Predicted (42 ± 18.8), without hyperinflation (TLC % Predicted 107 ± 24.8). 40 (71%) patients had structural evidence of emphysema on chest CT imaging. The mean number of moderate exacerbations in the year prior to initiation of biologic therapy was 3.63 ± 2.6. The mean number of severe exacerbations in the year prior was 1.34 ± 1.92. The mean eosinophil count was 414 ± 296 cells/µL.

### Glucocorticoid utilization

**Fig. 1:**
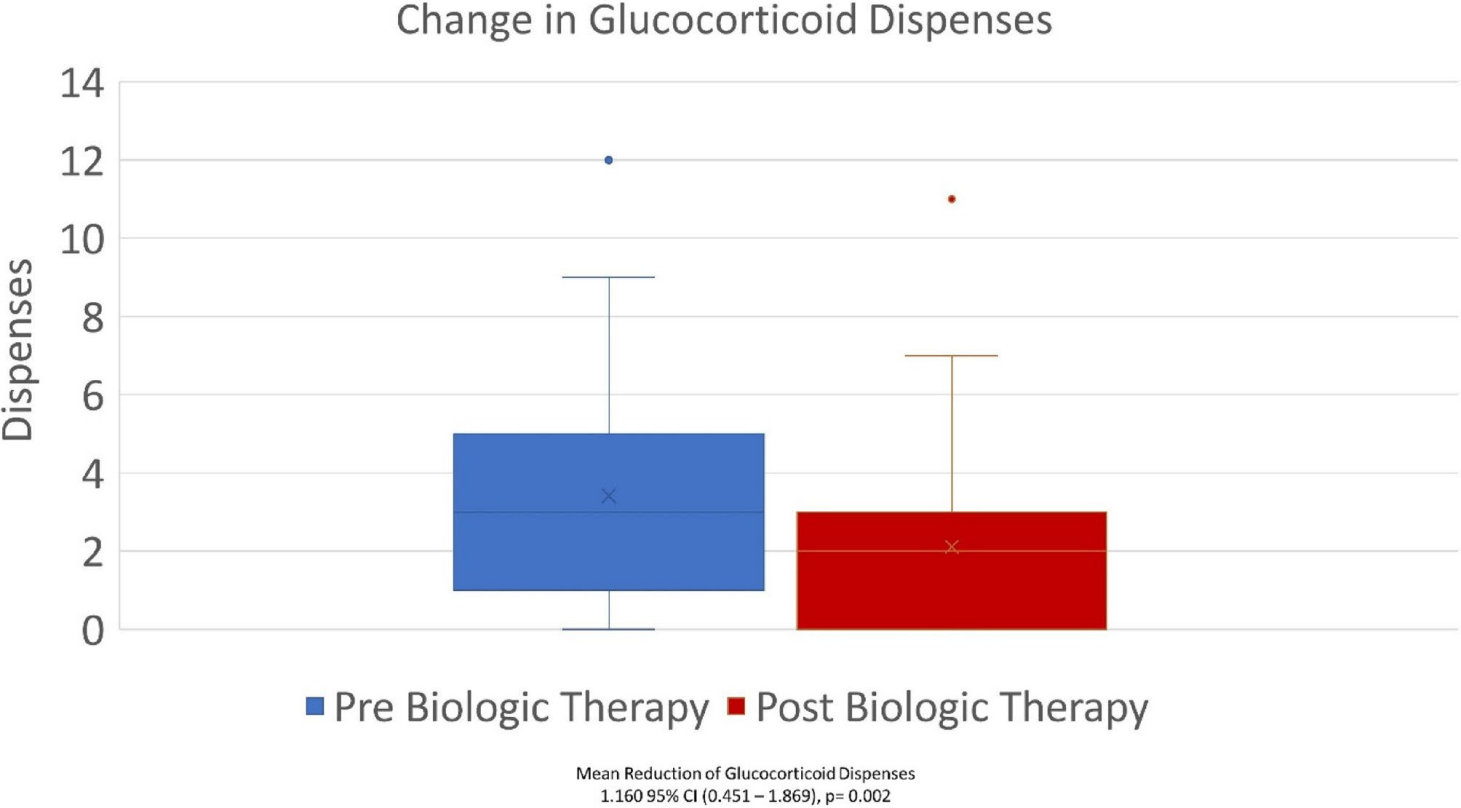
Change in Glucocorticoid Dispenses Pre and Post Biologic Therapy

**Fig. 2:**
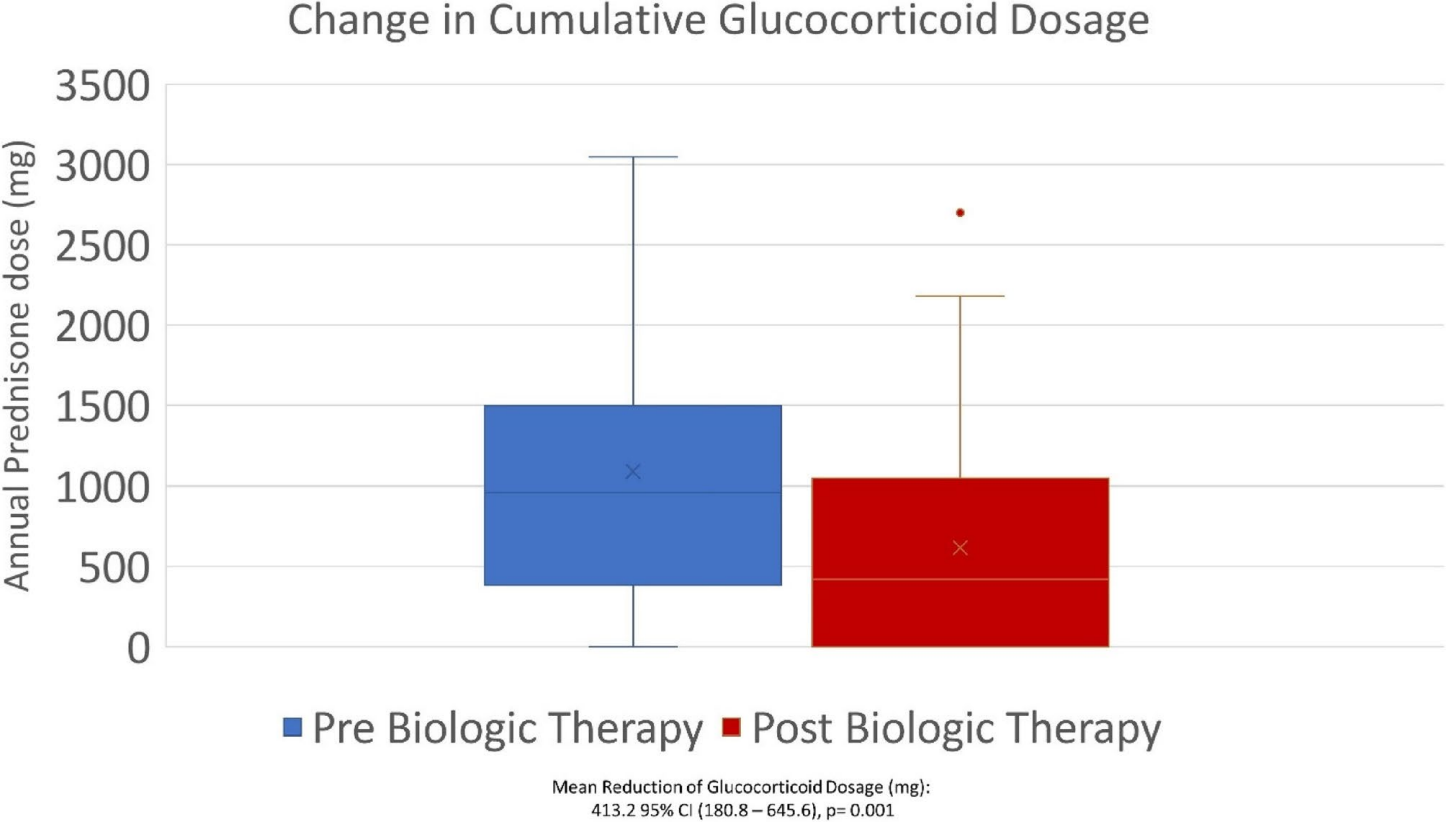
Change in Cumulative Glucocorticoid Dosage (mg of prednisone) Pre and Post Biologic Therapy

Glucocorticoid utilization, in terms of dispenses and total yearly dosage, were both significantly decreased following initiation of biologic. The total number of yearly systemic glucocorticoid dispenses decreased from 3.38 ± 2.58 to 2.22 ± 2.33, with a mean reduction of 1.160 dispenses (95% CI 0.45–1.87, *p* = 0.002) (Fig. 1). Cumulative yearly dosage of glucocorticoids, measured in mg of prednisone decreased from 1073 ± 831 to 659 ± 723, with a mean reduction of 413.2 mg (95% CI 180.8–645.6, *p* = 0.001) (Fig. 2).

**Fig. 3:**
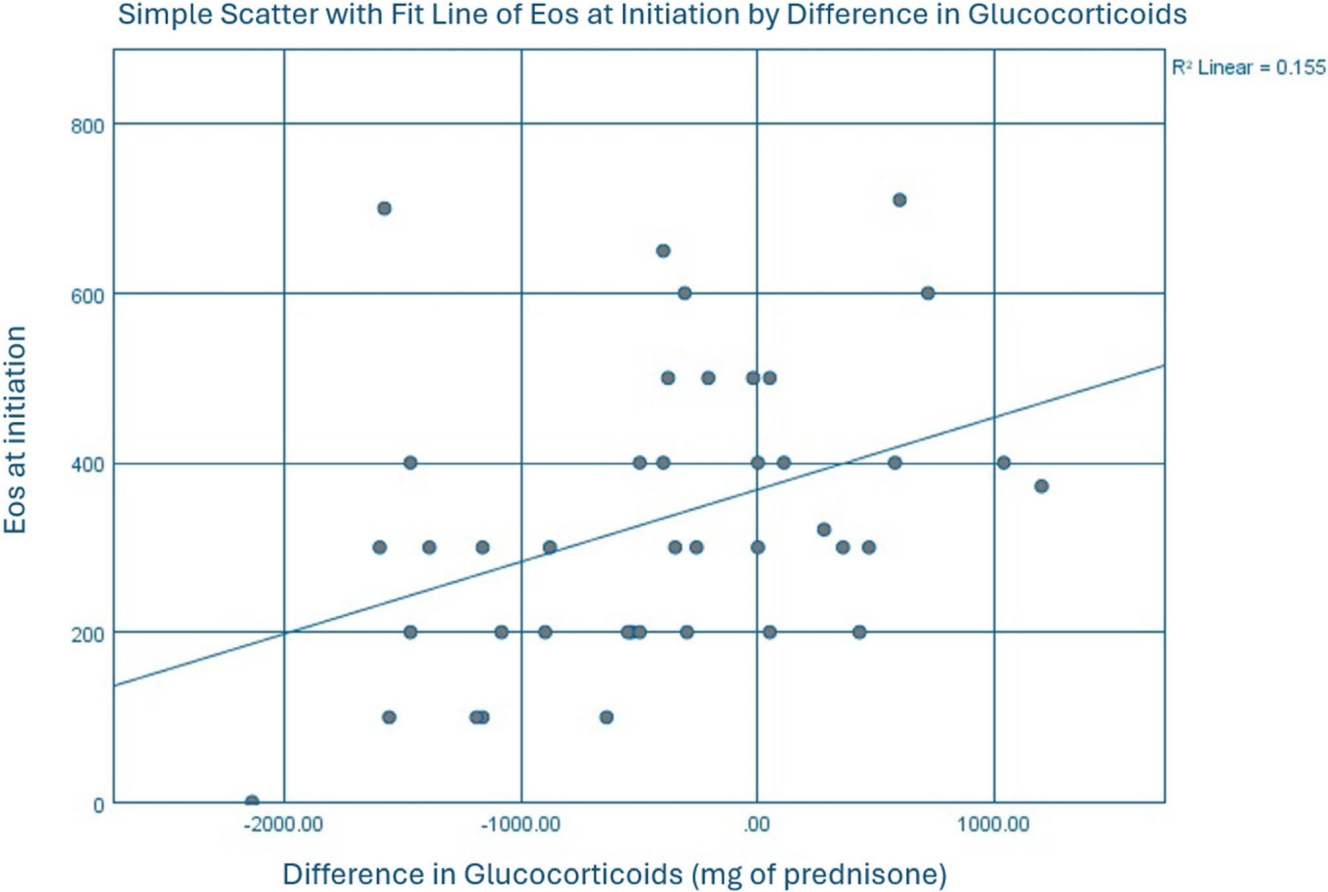
Relationship between Eosinophil Count at Initiation and Change in Glucocorticoid Dosage

Linear regression analysis was performed to evaluate the relationship between eosinophil count on initiation of biologic therapy and metrics of glucocorticoid utilization. The analysis showed a weakly positive association between baseline eosinophil count and change in glucocorticoid dispenses, *R* = 0.384, *p* = 0.008 and baseline eosinophil count and change in cumulative glucocorticoid dosage, *R* = 0.394, *p* = 0.011 (Fig. 3).

## Discussion

Utilization of biologic therapy in our cohort of real-world COPD patients with T2 inflammation showed significant decreases in overall systemic glucocorticoid utilization. This was reflected in both the total cummulative dosage, and the frequency of dispenses. On average, our patients were able to reduce the number of dispenses by 1.16 per year, along with a mean reduction of 413 mg of prednisone per year. These findings are significant as this is the largest study, to our knowledge, that shows that the use of biologics can reduce systemic glucocorticoid utilization in a real-world cohort of patients with COPD.

Reducing systemic glucocorticoid usage in patients with COPD is important, again, due to the significant side effects associated with even short courses [2, 4, 5]. The ability of biologics to reduce systemic glucocorticoid utilization has been extensively studied in asthmatic patients. In the SIRIUS trial from 2014, investigators assessed the impact of mepolizumab in patients with severe eosinophilic asthma and found that treated patients were 2.39 times more likely to have a reduction in glucocorticoids, with a 50% median reduction in baseline glucocorticoid dose [16]. These steroid-sparing findings were again shown in the benralizumab and dupilumab trials, with overall dose reduction rates up to 75%, with a significant number of patient coming off of oral corticosteroids completely [15, 18, 20].

A dose response curve has also been proposed in asthmatic patients, in which significant adverse outcomes present at thresholds as low as 500-1000 mg (prednisolone equivalents) of lifetime exposure [21]. These are significant adverse events including diabetes, depression, renal impairment and osteoporosis. In our cohort, the mean cumulative prednisone dose prior to biologic therapy was 1073 mg, which decreased to 659 mg after treatment, representing a reduction to 61.4% of their baseline dosage. Notably, the average patient in our study has already exceeded the 500 mg threshold, in just one year, placing them at elevated risk of glucocorticoid associated side effects. The potential degree by which glucocorticoids can be reduced in COPD patients, as compared to what was described in asthmatics, remains to be seen and warrants further investigation.

There was a weak, but significant association between eosinophil count at baseline and both increased cumulative glucocorticoid dosage, *R* = 0.394, *p* = 0.011, and increased glucocorticoid dispenses, *R* = 0.384, *p* = 0.008, which was surprising as we hypothesized that those with higher eosinophil counts would have better responses to biologic therapy. Some of this may be attributable to the retrospective design of the study. The timing of blood draws was not protocolized, not collected in relationship to exacerbations, and patients who recently received glucocorticoids could have lower eosinophil counts. These patients, therefore, despite having lower eosinophil counts, could represent a sicker cohort of patients that derived greater reductions in glucocorticoids with biologic therapy. Eosinopenia during COPD exacerbations has been associated with increased inpatient mortality along with other markers of acute illness, providing a rationale for this theory [22]. There is also data that suggests that eosinophils, while surrogates of T2 inflammation, are not the only considerations in the prediction of COPD exacerbations [23, 24]. Overall, the positive association of peripheral blood eosinophilia and increased glucocorticoid utilization was weak but requires increased investigation in prospective studies.

Limitations include the retrospective nature of the study which may introduce selection bias into our cohort. In addition, the sample size is relatively small, however there are limited studies regarding real-world COPD patients treated with biologics and our study provides data regarding an important and unstudied outcome of therapy. Glucocorticoid usage was also only quantified by exacerbation doses of prednisone. Based on retrospective review of the electronic medical record, the documentation of glucocorticoid usage of patients on chronic long-term doses was unreliable and therefore was not included. We believe that our data still captures the most relevant usage of glucocorticoids, those associated with exacerbations, without compromising the findings, given the inconsistent documentation of duration of chronic glucocorticoids. It is theoretically possible, but much less likely, that patients who were on long term glucocorticoids had increased usage in the year after initiation of biologics. It would be more likely that this group showed improvement as well, given the data in steroid reductions that have been demonstrated in asthmatic patients, but this was not able to be assessed in our study. While biologics were not approved for the treatment of COPD during the study period, emerging evidence at the time supported off label use of biologics in patients with T2 high inflammation [13, 14]. In addition, patients continued to have exacerbations despite optimal medical therapy. Different biologic agents were used in our patients but were not compared to one another given the small sample size and retrospective design of the study. 

The ability to completely avoid potential asthmatics in our cohort remains a consideration. This is an important caveat, however these patients had significant smoking histories (mean 47 pack years), imaging findings of emphysema (71%) and the diagnosis was established by an attending pulmonologist. Carrying a diagnosis of asthma is common and misclassification of patients happens often [25]. Patients with dyspnea are commonly treated with inhaled bronchodilators despite not having a diagnosis of obstructive lung disease [26, 27]. Due to the fact that biologics were not approved for COPD as a diagnosis at the time of the study, patients may have been diagnosed with Asthma-COPD overlap syndrome, whereas currently, they could be described as COPD with type 2 inflammation. Taking this into consideration, we believe the patients in our study reflect a real-world population where COPD diagnoses are often less clearly delineated than in the controlled settings of clinical trials.

The findings of our study add to the accumulating evidence that shows that biologics have a role in select patients with COPD. In addition to reducing exacerbations, the ability to reduce overall glucocorticoid usage is encouraging, especially due to the associated side effects in a patient population that has a high comorbidity index of obesity, osteoporosis and frailty [28]. While there are strong associations between glucocorticoids and adverse effects, future prospective studies should ideally evaluate if biologic therapy can mitigate not only the glucocorticoid dose but also the adverse events, such as fracture risk, diabetes, and neuropsychiatric symptoms.

In conclusion, in patients with COPD and evidence of T2 inflammation, the usage of biologics was associated with a statistically significant and potentially clinically relevant reduction in systemic glucocorticoid exposure.

## Data Availability

The data that supports the findings of this study are available from the corresponding author upon reasonable request.

## Abbreviations

COPD: Chronic Obstructive Pulmonary Disease
mg: milligram
PFT: pulmonary function tests
T2: Type 2
